# Understanding nurses’ experiences with near‐miss error reporting omissions in large hospitals

**DOI:** 10.1002/nop2.827

**Published:** 2021-03-03

**Authors:** Jaehee Lee

**Affiliations:** ^1^ Department of Nursing Sehan University Yeongam‐gun Korea

**Keywords:** near‐miss error reporting, near‐miss errors, phenomenological qualitative research

## Abstract

**Aim:**

This qualitative study aimed to provide an in‐depth understanding of nurses’ experiences with near‐miss errors and report omissions known to be direct or indirect causes of medical accidents in hospitals and cited as precursors of serious medical accidents.

**Design:**

This study collected experiences of research participants through an interview as a qualitative research method and confirmed the meaning through an inductive approach.

**Methods:**

We selected nine nurses with various levels of experience from 27 May to 10 June 2019 for analysis. We adopted phenomenological research methods and procedures proposed by Colaizzi (Existential‐phenomenological alternative for psychology, 1978) and established the feasibility and integrity of our results based on narrative studies proposed by Lincoln and Guba (Naturalistic inquiry, 1985).

**Results:**

This study demonstrated that near‐miss errors and report omissions experienced by professional nurses could be merged into the following themes: lack of cognitive susceptibility to near‐miss errors; confusion about the reporting system for near‐miss errors; lack of knowledge about near‐miss errors; disappointment with results of reporting near‐miss errors; and fear of reporting near‐miss errors. These results strongly suggest the need to improve recognition efforts based on a socio‐educational viewpoint involving the so‐called openness about failures.

**What does this paper contribute to the wider global clinical community?**

- This study suggests that sharing the process and results of near‐miss errors in clinical field sites will provide an important foundation in securing the safety of patients.
- This study suggests that sharing the process and results of near‐miss errors will contribute to the development of not only individual nurses but also the whole hospital community.
- This study suggests that sharing the process and results of near‐miss errors by openly acknowledging those mistakes will provide the best learning opportunity and serve as a cornerstone for improving clinical practice.

## 1 INTRODUCTION

Near‐miss errors are errors that occur to a patient. However, the patient is never harmed because of fortuitous or appropriate intervention (Capucho, 2011; Kim, 2006). Although damage has not occurred yet, such errors are highly likely to cause a damage. Near‐miss errors are also referred to as potential adverse events (Capucho, 2011). Near‐miss errors are invisible. However, medical accidents are clearly visible. If near‐miss errors are never addressed internally or debated publicly, it is nearly impossible to discuss contexts related to near‐miss errors.

Despite remarkable progress has been made for skill levels of medical staff and medical technology, there has not been significant progress in reducing the occurrence of medical accidents and subsequent damages (Asaoka et al., 2013; Kim et al., 2006). As it is difficult for patients to recover both physically and psychologically from serious medical accidents, prevention before occurrence is of paramount importance. With this recognition, near‐miss errors are attracting much attention as precursors of medical accidents. Specifically, reducing cases of these errors is perceived as effective for preventing medical accidents (Lee, 2012). In fact, it is thought that near‐miss errors occur approximately 7,700 times as often as serious medical accidents (Wagner et al., 2006). Considering that some eligible cases are not omitted in the recognition process of medical accidents, there might have been more near‐miss errors than the number assumed (Kim et al., 2007). However, as most of existing studies have used quantitative approaches (Elder et al., 2007; Kim et al., 2007; Lee et al., 2013; Mayo & Duncan, 2004) in targeting sample groups as the mainstream research method, it is difficult to sense the seriousness of the situation intuitively. Moreover, the limited number of question items makes it impossible to develop an in‐depth level of understanding. In particular, a limitation of existing studies is that they provide only a superficial understanding of experiences of medical accident victims. Thus, the purpose of this study was to provide detailed descriptions of experiences and perceptions of persons involved in near‐miss error reporting omissions based on a qualitative approach in pursuit of a naturalistic paradigm. Given this, findings of this study are meaningful as basic data for various related future studies.

## 2 CONCEPT OF NEAR‐MISS ERRORS

Near‐miss errors are errors that occur to a patient. However, the patient is never harmed because of fortuitous or appropriate intervention. Although damage has not occurred yet, such errors are highly likely to cause damage. Near‐miss errors are also referred to as potential adverse events (Capucho, 2011). Near‐miss errors are invisible. However, medical accidents are clearly visible. If near‐miss errors are never addressed internally or debated publicly, it is nearly impossible to discuss contexts related to near‐miss errors objectively, which is a mainstream discussion method. Data of true contexts of near‐miss errors as precursors to medical accidents are needed.

## 3 STUDY DESIGN

This study collected experiences of research participants through an interview as a qualitative research method and confirmed their meanings through an inductive approach. Specific research methods are shown below.

## 4 METHODS

### 4.1 Participants

Participants of this study were nine nurses working in large hospitals in South Korea. Three of these nurses had 2 to 4 years of career experience, and six of them had 10 to 13 years of career experience. All nine nurses had either directly or indirectly experienced near‐miss errors and reporting omissions at their hospitals. A study cooperation letter was sent to hospitals of these participants. Nine voluntary participants were selected based on gender, region, education level and career.

### 4.2 Data collection

Original data for this study were collected using an individual in‐depth interview method as proposed by Colaizzi (1978). Interviews were recorded, and data were collected together with field notes. In the first round of individual interviews with study participants, we tried to build a mutual rapport through wide‐ranging conversation concerning the purpose, intent and topic of this study. Beginning with the second round, snowball interviewing was conducted by developing stories based on the first round of interviews and creating additional questions (Heo et al., 2019; Kim, 2005). This study involved a total of 14 rounds of interviews, after which data reached theoretical saturation as no new information was revealed. Five of these 14 sessions were conducted in the form of group interviews, while nine were conducted through individual interviews. These interviews were conducted in researchers’ laboratories for five months, from 10 January 2019 to 27 May 2019.

### 4.3 Data analysis and research authenticity

Data analysis was conducted at the same time as data collection. Data were analysed based on the six‐step analysis method suggested by Colaizzi (1978). First, after completing the transcription of collected data, the researcher repeatedly read all statements to identify and understand the phenomenon. Second, meaningful statements were derived by underlining phrases and sentences through line‐by‐line analysis. Third, a restatement with a general form was conducted from a meaningful statement. Fourth, an attempt was made to identify similar statements at the stage of deriving the meaning formed from the meaningful statement and the restatement. Fifth, after integrating analysed contents, a final description was made through a collection of topics and categories that symbolized the essential structure of the research phenomenon. Sixth, an attempt was made to describe the phenomenon as accurately as possible with a complete description representing the essential structure. The most appropriate statement describing the topic as a quotation was then selected.

In this study, the entire information obtained from data collection and analysis process was shared with study participants. The researcher performed this to minimize errors arising from misunderstanding or misinterpreting participants' stories or statements. This study did not rely only on interviews to collect data, but widely used related document records and journals written by study participants. The validity of all research processes, analysis of collected data, content analysis and subject development were verified by two professors in the nursing department, two doctoral researchers with extensive qualitative research experience and two nurses with more than 10 years of experience in large hospitals. In general, this series of methods to increase the veracity and validity of research results in qualitative studies is called triangulation which can increase the transferability of results (Lincoln & Guba, 1985).

### 4.4 Ethical considerations

Before initiating this research, participants were fully informed about the intent and purpose of this study. They were also informed that whenever they wanted to know study contents, the information would be provided to them. Participants were informed that they could leave the study at any time and that all information acquired in the research processes would be used only for research purposes. Approval was obtained from our Institutional Review Board before initiating the research. In addition, only participants who agreed to participate were included in this study. All participants were anonymous.

## 5 RESULTS

### 5.1 Participant demographics

There were nine nurses as participants (seven females and two males). Six nurses worked at large hospitals in Seoul and three worked in Gyeonggi‐do Province. In terms of their academic backgrounds, five nurses had graduated from nursing colleges and the remaining four held four‐year degrees. Table 1 presents demographic characteristics of study participants. Themes identified from interview responses of these nine nurses through a data collection process are presented in the following sections.

**TABLE 1:** Demographic characteristics of participants

Participant	Sex	Location	Education	Total Clinical Experience (years)
A	Female	Seoul	College	13
B	Female	Gyeonggi‐do Province	College	2
C	Male	Seoul	College	10
D	Female	Seoul	College	10
E	Female	Seoul	Bachelor	11
F	Male	Gyeonggi‐do Province	Bachelor	12
G	Female	Gyeonggi‐do Province	College	3
H	Female	Seoul	Bachelor	4
I	Female	Seoul	Bachelor	12

### 5.2 Theme 1. Lack of cognitive susceptibility to near‐miss errors

Based on responses, it was found that study participants did not notice that they made near‐miss errors. In other words, they did not have near‐miss error sensitivity. They did not realize the seriousness of such errors or they did not report them. None of them had ever felt an urgent need to address such errors. Study participants perceived near‐miss errors as part of a mechanical routine. They even considered such errors as natural mistakes. They said that they did not report near‐miss errors by ignoring them, treating them as they had always been treated and justifying them as everyday errors. In terms of cognitive susceptibility, these nurses perceived near‐miss errors not as malicious, but as part of their daily job routines (Table 2).

In the early days of my career, I did not know which mistake was a serious one or a minor one, and I just did what I was told to do. At that time, there was a preceptor who would take responsibility. However, when I became a senior nurse, these problems that I had overlooked or habitually done were not big things, and so I did not think it was necessary to report them. This was the way I worked. (Nurse I, 12 years of career experience)

There are no clear criteria to tell which mistake is big or small. I just did it as I was instructed to do, so it was not my fault. For instance, when a patient complained of indigestion after taking a drug that was routinely administered, a senior nurse told me that as the drug was administered without causing any problems, it was highly unlikely that it was a side effect. So, I thought it was not necessary to report a minor side effect. The senior nurse's word is a kind of law. (Nurse B, 2 years of career experience)

It is very exhausting to deal with patients in the outpatient ward. This story occurred a year ago. If patients have the same name, I usually check their dates of birth. On that day, I just saw the name but failed to check the date of birth. As a result, I was looking at the treatment history of another patient. I did not instruct the staff (a patient manager in front of the treatment room) to check the patient's date of birth because we had an outpatient with the same name that day. (Nurse C, 10 years of career experience)

**TABLE 2:** Nurses’ experiences and reporting omissions of near‐miss errors

Themes	Theme‐cluster	Category
Too small and a small mistake	Ignore errors	Near miss/Lack of cognitive sensitivity
Happens all the time		
Is this a mistake?		
Everyone makes mistakes in the beginning		
A small mistake can be hidden		
There is nothing wrong	Error inertia	
Nothing ever happened		
Things like that happen		
So what?		
Happening	Routine error	
Tired and tired daily		
Do it every day		
It is awkward when it is suddenly different		
It has happened before		
Who do you report to?	Subject of report	Near miss/Reporting system confusion
Which department gets the report?		
How do you report it?	Report procedures	
What do you need to report?		
What is the reporting procedure?		
What do you report?	Report contents	
Whose mistake does it report?		
If there are multiple or complex errors, how do I report them?		
How do you organize your report?		
I did what you said		
Does it make a difference if I report it?	Effect of reporting	Near miss/Disappointing reporting effect
Mistakes continue even after reporting		
No contact after reporting	Reporting feedback	
To whom did my report go?		
Why am I the only one who reports?	Unfairness	Near miss/Fear of reporting
<The other person did not report anything worse>		
Why are you reporting this one?		
<I did not report it before>		
Why do you report this?		
<Unfairness of formal report>		
Do you know how long my career is? You embarrass me.	Humiliation	
Embarrassed to know		
There is nothing good to report		
Implicit disadvantage	Disadvantage	
Official disadvantage		
I did not learn this in school	Absence of knowledge and information	Near miss/Lack of knowledge
I know roughly what it is.		
That is the first time I have heard it since I joined the company		
I did not know our hospital had a near‐miss error reporting system		
Is reporting important?	Absence of attitude	
I will be careful next time		

### 5.3 Theme 2. Confusion about the reporting system for near‐miss errors

Study participants’ confusion about reporting near‐miss errors took two main forms. One, nurses in this study discussed the lack of clear guidelines about who should report near‐miss errors and to which department or institute they should report them because there were no clear written or oral reporting procedures at their hospitals. Two, these nurses mentioned that there was no clear definition of a near‐miss error. There were no contexts or guidelines for distinguishing one's own mistakes from those of others’, attributing causes of errors, or clear delineation of responsibility in the event of multiple errors. The lack of clear guidelines is a visible factor. However, the latter factor is invisible. All study participants reported that both visible and invisible factors led to their confusion about near‐miss errors, making it difficult for them to know how to report them and which caused reporting omissions (Table 2).

I do not know exactly to whom I have to report when a near‐miss error occurs. I do not know whether I have to report to my immediate boss or a person in a higher position. I do not know what I have to prepare to report it. My heart trembles, which makes me feel the situation even harder. And I do not know to whom I can report it to or to whom I can talk about it. (Nurse G, 3 years of career experience)

Whenever a near‐miss error occurs, I feel scared and burdened. When a near‐miss error happens, I feel alone. It seems that no one can help me out and I feel lonely. I talked about it to one of my colleagues one day, but it ended up in miscommunication, which put me in a more difficult situation. (Nurse H, 4 years of career experience)

### 5.4 Theme 3. Lack of knowledge about near‐miss errors

There is an acknowledged difficulty in articulating clear definitions of types and scope of near‐miss errors that occur in hospitals or clear responsibilities for such occurrence. However, nurses need to have basic knowledge about the concept of near‐miss errors including the importance of both countermeasures and reporting in the event of near‐miss errors. In this study, the lack of this conceptual knowledge played a major role in study participants’ experiences with near‐miss errors and reporting omissions.

For example, beginner nurses in this study were unaware of the term “near‐miss errors” and its usage before they joined their hospitals. On the contrary, nurses with many years of career experience who were aware of near‐miss errors and reasons for reporting still did not feel the need for such reporting. It can be hypothesized that all practices derive from the recognition of problems and unawareness of near‐miss errors and their importance will decrease the probability of reporting. Nurses who do not perceive such errors will not think or know to report about them (Table 2).

I heard the word of “near‐miss error” for the first time after joining the hospital. I might have heard the term during a preliminary nursing curriculum, but the concept is something I do not know very well. (Nurse B, 2 years of career experience)

I do not remember that I learned the word “near‐miss error” when I was in college. I do not think that the term has ever appeared in any exam. After entering the hospital, senior nurses provided many different instructions concerning work. I have learned the procedures and methods to perform those instructions without making mistakes. When I made a mistake, I have never been told by anyone whether it was a near‐miss error or not. In fact, I know that a near‐miss error is a minor error, but I do not know the exact concept or meaning. (Nurse H, 4 years of career experience)

The operating room is divided into a strict sterile field and a non‐sterile field. Because they are not visibly divided, I do not think seriously when I see a contaminated thing inside a non‐sterile field. If I think of it now, it is the most serious thing that could have happened. I did not understand whether that was a near‐miss error or not. Of course, I did not report it. (Nurse C, 10 years of career experience)

When I checked my patient’s vital signs one day in the early period of my career, I made a mistake of forgetting to check the blood pressure. So, I just filled in a similar number without taking an actual measurement. The next day, the patient's blood pressure went up too high and I had to report it. I made an excuse by saying that I did not see any abnormality when I checked it the day before. So, is this a near‐miss error. (Nurse G, 3 years of career experience)?

### 5.5 Theme 4. Disappointment with results of near‐miss error reporting

Nine participants had experiences of reporting formally and informally about near‐miss errors and omission of reporting. Study participants were sceptical about how a systematic and detailed reporting of routine and minor mistakes such as near‐miss errors could contribute towards the prevention of actual safety accidents. These participants said that irrespective of their direct or indirect experiences with near‐miss error reporting, similar errors continued to happen. Moreover, because immediate measures such as education and information sharing did not occur frequently before, they doubted effects of a near‐miss error reporting. In addition, these nurses perceived reporting to be meaningless because when they had reported such errors, they had never been informed about the progress of any follow‐up measures even when they had submitted detailed reports about such incidents. These experiences had eventually led participants to think that reporting near‐miss errors would fall on deaf ears, which in turn gave nurses negative impressions about near‐miss error reporting. These nurses’ perspectives within this theme indicated low perceptions of the value of noting and reporting near‐miss errors (Table 2).

Is it because there is no disadvantage caused by a near‐miss error? Even after reporting a near‐miss error, I did not hear that any measures were taken to address the issue. (Nurse F, 12 years of career experience)

Even when a near‐miss error was reported after its occurrence, I did not remember how it was handled, and I did not think I received any education about it. It might have been handled through a non‐official route, or it might have been discussed personally with the concerned person, but there were no follow‐up measures. Therefore, I feel I am just making a fuss out of nothing, so I just skip reporting it. (Nurse C, 10 years of career experience)

Maybe I made a mistake because I felt tired due to overwork the previous day. I did not know exactly what kind of mistake I made and hesitated to report it. If I had reported it, I should have been informed about the cause and the effect of the near‐miss error, but that was not the case. (Nurse A, 13 years of career experience)

It might not be a problem if there is a clear definition of responsibility. But if several people are involved, I am concerned whether I might be more disadvantaged than others. Of course, I doubt whether the report could represent me well or not. In most cases, people tend to support those who are closer to them. (Nurse H, 4 years of career experience)

### 5.6 Theme 5. Fear of near‐miss error reporting

A near‐miss error is an obvious mistake, whether it is officially reported or not. Therefore, individuals have a psychological and emotional repulsion of mistakes made during the performance of their work. Nurses often avoid reporting near‐miss errors because they are responsible for their own mistakes. In interviews of this study, nurses confessed that they deliberately ignored and avoided these responsibilities and explained that this was due to disadvantages of reporting near misses (Table 2).

I made a mistake because I felt extremely tired due to overwork the day before. It might sound like an excuse. I am afraid that people might think I made a mistake not because I was too tired but because I am incompetent. I doubt whether my situation can be properly represented. (Nurse F, 12 years of career experience)

When I make a near‐miss error, I might openly admit it as my own. Still, I cannot imagine what kind of disadvantages or penalties I would have to undergo after that confession. It is a really scary thing to experience. I might have to endure salary reduction or other disadvantages. What is scarier than this is what other peer nurses might think of me. There are a lot of things to worry about. I am worried about how my subordinates think of me. I am also afraid that I might not get promoted because of that mistake. (Nurse H, 4 years of career experience)

If one of my colleagues makes an unpleasant comment when I make a near‐miss error, or if I make the same displeasing comment when my colleague makes a mistake, either case can hurt my relationship with the colleague. Sharing the responsibility for near‐miss errors is actually like sharing one’s own mistakes, but it is not easy to admit one’s own mistake and share everything about it. If we share information about near‐miss errors, we probably will not repeat the same mistakes. But it is not easy. I do not think it will work unless we reveal everything about what have happened. (Nurse I, 12 years of career experience)

## 6 DISCUSSION

Results of this study are as follows. Study participants’ experiences with near‐miss errors and omissions of reporting near misses were due to the following: (a) lack of near‐miss sensitivity, (b) confusion regarding near‐miss reporting systems, (c) lack of near‐miss knowledge, (d) distrust of near‐miss reporting effects and (e) fear of near‐miss reporting. First of all, lack of near‐miss sensitivity, confusion regarding near‐miss reporting systems and lack of near‐miss knowledge have different contents and circumstances. However, they are all due to ignorance. Because of this ignorance of near misses, study participants have accepted these events as part of their daily routine. These participants’ ignorance of near‐miss acts was manifested as a distrust of near‐miss reporting effects, leading to further distrust of the importance of thorough reporting, information sharing, and the effect of reporting. That is why these nurses do not feel the need to report minor near misses, leading to the fear that near‐miss reporting will result in actual and implied disadvantages. In this study, the experience of omitting near‐miss reporting was not limited to one factor, but was caused by multiple factors. The experience of a near miss and “the omission of near‐miss reporting” stated by nurses who participated in the study reflected results of a previous study that reported that 76.5% of hospital nurses did not report near‐miss errors (Kim et al., 2006).

According to previous studies, causes of reporting omissions can be summed up as follows: nurses perceive near‐miss errors as simple mistakes that are harmless to patients (Kim et al., 2006; Kim, 2006), there is a lack of knowledge about accurate criteria for error detection (Kagan & Barnoy, 2008), there is a fear of possible penalties and blame (Elder et al., 2007; Mayo & Duncan, 2004), there is a lack of confidence about improvement effects and there is concern that the information will be used to evaluate job performance (Ahn et al., 2007). Other reasons for the low rate of reporting near‐miss errors include a lack of confidentiality, a lack of time, no feedback on reported cases (Kaplan & Barach, 2002), a fear of peer or boss reactions (Mayo & Duncan, 2004) and a negative attitude towards near‐miss error reporting (Kim et al., 2006). With these justifications and explanations, it is possible that the potential danger of near‐miss errors is being downplayed because nurses cannot establish a negative impact of these near misses on their patients (Cohen, 2000). Thus, it is natural that most of related previous studies on this topic are discussions about methods for improving the phenomenon of not identifying and reporting near‐miss errors in hospitals.

The proverbs and adages about mistakes in Western and Oriental worlds commonly say that human deeds are inevitably premised on mistakes and failures. They provide consolation that humans learn lessons from their failures and mistakes to pursue new success and perfection. This viewpoint of leveraging individual mistakes and failures to improve practice has attracted much attention in the discourse of educational sociology. From a behavioural perspective, because failure interferes with performance and triggers negative emotions such as frustration and helplessness, individuals will try to avoid the experience of failing (Bartels & Ryan, 2013; Mullet et al., 2014).

From the viewpoint of a supervisor, it is inevitable to control errors and failures which can be considered antagonistic to success. However, for the performer, the only two choices are failure and success. In that context, mistakes that do happen can be concealed. Accumulated hidden mistakes can eliminate opportunities for improvement. Such failure to improve is highly likely to lead to learned unconcern which is related to learned helplessness (Nanda et al., 2012). After all, the perception of the learning process (Bauer & Mulder, 2007; Cha & Cho, 2016) or the perspective of constructivism (Son, 2005) that leads to professional improvement and progress derives from disclosing errors and mistakes for better understanding between related parties.

In this regard, disclosure of academic failure (Cannon & Edmondson, 2005; Kim, 2017; Lee, 2018) is based on the belief that failure is not necessarily negative because it can ultimately lead to a positive practice. This belief strongly emphasizes the mutual benefit of disclosing failure and offering assistance in turn. People who are open‐minded about failure can easily obtain information they need and share their experiences with peers and colleagues. Disclosing errors also facilitates adaptive emotional responses, motivates active learning and ultimately provides a foundation for positive achievement (Kim, 2017; Lee, 2018). With the above as our guide and in the context of the topic of reporting near‐miss errors in hospitals, we strongly recommend disclosing information about these errors to relevant people to increase awareness and prevention before such errors cause damage to patients.

## 7 CONCLUSION

Up to date, discussions on near‐miss errors have mainly focused on identifying their causes and substantially reducing them. Researchers have also discussed near‐miss errors from a more macroscopic viewpoint (Lee et al.,^2008^, ^2013^; Jeong et al., 2006; Park et al., 2006) and presented methods to establish and utilize hospital systems to prevent medical accidents by promoting near‐miss error reporting. However, the presentation of near‐miss errors as failures to be avoided is consistent across studies.

On the contrary, we propose sharing the process and results of near‐miss errors. Also, it is inferred that disclosing failure is the best learning opportunity for improving practices as a cornerstone for further progress. If near‐miss errors become perceived as opportunities for individual and institutional development rather than as failures, they can prompt active and lively discussions on previously hidden near‐miss error cases. Such changes represent a foundation for mutual development of nurses and hospital communities and for patient safety.

## 8 LIMITATIONS OF RESEARCH

This study has the following limitations. First, because this was a qualitative study and the main purpose of the study was to interpret subjective experiences of participants and discuss their meaning, it was difficult to generalize results of this study. Second, it was difficult to collect various cases because participants’ geographical areas in this study were limited to easily accessible areas.

## 9 RELEVANCE TO CLINICAL PRACTICE

To reduce near‐miss errors, clinical nurses must make efforts in the following three dimensions. First, negative impressions of near‐miss errors must be reframed and viewed as opportunities or cases to enhance nursing practice. Especially from the viewpoint of lifelong essentials for professionals like nurses, near‐miss errors are important issues that must be shared and learned. More concrete and practical efforts must be made for hospital cultures to perceive near‐miss errors as opportunities to learn lessons. Second, supplementary training should be provided to currently practising nurses to raise their awareness about the nature and importance of near‐miss errors and to systematically manage and distribute lessons learned from them. Lastly, a more systematic and effective transfer of knowledge about near‐miss errors is needed for the education of prospective nurses. In this study, nurses with relatively shorter career experience said that they had not heard the term “near‐miss error” until they started working for hospitals. This shows that education on near‐miss errors is rarely included in the curriculum for prospective nurses. Thus, an in‐depth discussion has to be undertaken regarding the reestablishment of empirical and theoretical systems for near‐miss errors and incorporation of them into nursing curriculum.

## CONFLICT OF INTEREST

No conflict of interest has been declared by the authors.

## Data Availability

The data that support the finding of this study are available from the corresponding author upon reasonable request.
